# Physiological assessment of a 16 day, 4385 km ultra‐endurance mountain bike race: A case study

**DOI:** 10.1113/EP091260

**Published:** 2024-01-08

**Authors:** Robert D. Hyldahl, Jayson R. Gifford, Lance E. Davidson, Chad R. Hancock, Paul S. Hafen, Allen C. Parcell, Gary W. Mack

**Affiliations:** ^1^ Department of Exercise Sciences Brigham Young University Provo Utah USA; ^2^ Department of Nutrition, Dietetics & Food Science Brigham Young University Provo Utah USA

**Keywords:** endurance exercise, marathon, mitochondria, skeletal muscle, ultra‐endurance, vascular function

## Abstract

The Tour Divide (TD) is a 4385 km ultra‐endurance bicycle race that follows the continental divide from Canada to Mexico. In this case study, we performed a comprehensive molecular and physiological profile before and after the completion of the TD. Assessments were performed 35 days before the start (Pre‐TD) and ∼36 h after the finish (Post‐TD). Total energy expenditure was assessed during the first 9 days by doubly labelled water (^2^H_2_^18^O), abdominal and leg tissue volumes via MRI, and graded exercise tests to quantify fitness and substrate preference. Vastus lateralis muscle biopsies were taken to measure mitochondrial function via respirometry, and vascular function was assessed using Doppler ultrasound. The 47‐year‐old male subject took 16 days 7 h 45 min to complete the route. He rode an average of 16.8 h/day. Neither maximal O_2_ uptake nor maximal power output changed pre‐ to post‐TD. Measurement of total energy expenditure and dietary recall records suggested maintenance of energy balance, which was supported by the lack of change in body weight. The subject lost both appendicular and trunk fat mass and gained leg lean mass pre‐ to post‐TD. Skeletal muscle mitochondrial and vascular endothelial function decreased pre‐ to post‐TD. Overall, exercise performance was maintained despite reductions in muscle mitochondrial and vascular endothelial function post‐TD, suggesting a metabolic reserve in our highly trained athlete.

## Abstract (highlights)

- **What is the main observation in this case?**
  This paper provides a unique multi‐parameter physiological analysis of a male subject who completed a 4385 km ultra‐endurance bicycle race. Although no specific hypotheses were tested, several intriguing observations were made. First, measurements of energy intake and expenditure indicated that energy balance was maintained, as was body mass. Second, after completion of the race, exercise performance was maintained despite reductions in skeletal muscle mitochondrial and vascular endothelial function.
- **What insights does it reveal?**
  Overall, the data provide a unique insight into the plasticity and tolerability of multiple physiological systems for the extreme physical stress imposed by multi‐day ultra‐endurance events in a trained athlete.

## 1 INTRODUCTION

Studies of outlier performance, extreme human phenotypes or feats of extraordinary endurance are capable of revealing important and fundamental physiological principles. For example, analysis of outlier world record performances by A. V. Hill in 1925 (Hill, 1925) has helped to influence our present understanding of muscular fatigue. Likewise, a recent study of Himalayan Sherpas has brought to light key metabolic adaptations that are likely to help shape our understanding of diseases that involve hypoxia (Horscroft et al., 2017), and a recent genetic study in a historically tall individual has contributed to our understanding of the genetic basis of height determination (Sexton et al., 2018). Along this same vein, we have taken advantage of the unique opportunity to perform a comprehensive molecular and physiological profile of a single individual before and after the completion of a 17 day, 4385 km ultra‐endurance bicycle event.

Recent studies have generally defined ultra‐endurance exercise in terms of either running more than 42.195 km (marathon distance) or cycling more than 100 miles (160.935 km). These studies have shed light on the nutritional and hydration requirements (Alcock et al., 2018; Belinchon‐Demiguel & Clemente‐Suarez, 2018) and, to a slightly lesser extent, the endocrine (Geesmann et al., 2017), immunological (Bellar et al., 2017) and metabolic (Coker et al., 2017) changes that accompany such efforts. For example, Plasqui et al. (2019) recently assessed the total energy expenditure (TEE) of seven male cyclists during a 24 day cycling race (Giro d'Italia) and found that total daily energy expenditure averaged ∼32.2 MJ/day (7719 kcal/day). Most of the published literature has focused on the requirements to succeed or perform at a high level in ultra‐endurance events, whereas many fewer studies have comprehensively interrogated the resulting cellular and molecular changes that result from participation in these types of efforts. Moreover, previous investigations have focused primarily on single‐day efforts.

In the present case report, we had the unique opportunity to profile the cardiovascular, metabolic, nutritional and body compositional changes that occurred in a single subject following the completion of the Tour Divide (TD), a unique, self‐supported mountain bike race that covered 4385 km and 60,000 m of elevation gain over a 16 day period.

## 2 MATERIALS AND METHODS

### 2.1 Tour Divide description and study design

The TD is a self‐supported mountain bike ultra‐race that begins in Banff, AB, Canada and finishes at the Mexican border crossing of Antelope Wells, NM, USA. The 4385 km route loosely follows the Continental Divide on dirt roads, double‐track and single‐track trails. The route includes ∼60,000 m of elevation gain. There are no aid or support stations, but the route travels through several small towns, where racers can resupply. Temperatures ranged from −3°C to 41°C over the entire route.

The subject participated in two data‐collection sessions. The first session occurred 35 days before the start of the TD. We felt that this time point was representative of the subject's typical trained state. The subject was asked not to train for 24 h before the session. The subject arrived at the laboratory in the morning having fasted for 8 h. The sequence of tests occurred as follows: resting metabolic rate, MRI, vascular function, blood sample, muscle biopsy and, finally, a graded exercise test. Blood (10 mL) was obtained by venipuncture from the antecubital vein, collected in serum vacutainers, and allowed to clot on ice for 10 min before centrifugation at 2000 *g* for 10 min. (BD Biosciences). After centrifugation, samples were placed in a −80°C freezer until being sent for analysis by the Utah Valley Outpatient Laboratory (Provo, UT, USA). Upon completing the TD at ∼14.00 h in Antelope Wells, the subject was driven to Grants, NM, USA (∼6 h), where he slept for ∼10 h. From Grants, the subject was driven back to his home in Utah (∼9 h). During travel, the subject consumed three large meals and frequent salty snacks and water. The following morning, the subject came into the laboratory fasted for the post‐TD measurement session. At this session, the subject underwent the same sequence of tests in the same order. The total elapsed time between finishing the TD and the post‐TD measurement session was ∼36 h.

The study conformed to the latest version of the *Declaration of Helsinki*. Data for this case study were not collected with any intent to test a hypothesis or otherwise produce generalizable knowledge and thus did not meet the common rule criteria for research [45 CFR 46.102(l)]. As such, the Institutional Review Board waived the need to give approval. Informed consent was provided in writing by the subject.

### 2.2 Muscle biopsy

A small area on the skin over the vastus lateralis was shaved, then cleaned with chlorhexidine. An injection of local anaesthetic (1% lignocaine with adrenaline) was used to numb the area. After the participant was able to report no sensation in the area, a small incision was made into the skin and fascia, and the biopsy needle was inserted into the muscle at ∼80 mm of depth. Using manual suction, ∼75–150 mg of tissue was withdrawn. The pre‐TD biopsy was taken from the right leg and the post‐TD biopsy from the left leg.

### 2.3 Body composition

Magnetic resonance images were obtained with a Siemens TIM‐Trio 3.0 T whole‐body scanner. A spin–echo sequence with a 525 ms repetition time and a 9 ms echo time was used for all acquired images, giving a 256 × 256 matrix within a 500 mm field of view. With the subject in a supine recumbent position, a T1‐weighted axial image (10 mm thick) was acquired at the L4–L5 intervertebral space and repeated every 50 mm (three images above and three below L4–L5) to quantify visceral fat, abdominal subcutaneous fat and abdominal/gluteal muscle. Leg muscle, intramuscular and subcutaneous fat were assessed by contiguous axial images (10 mm thickness, no interslice gaps) from the femoral head to the toes (∼110 images). SliceOmatic image analysis software (Tomovision, Montreal, QC, Canada) was used by a single image analyst to tag skeletal muscle, non‐muscle lean tissue and fat on each image. Tissue volumes, including the calculated interslice gap estimates in the abdomen, were converted to masses using assumed densities of 1.04 kg/L for muscle and 0.92 kg/L for fat.

### 2.4 Total energy intake and expenditure

During the TD, the subject maintained an audio‐recorded diet log, which was used to estimate daily calorie intake. To estimate energy expenditure during the ride, the subject was given doubly labelled water (^2^H_2_^18^O) on the day before the start of the ride. After providing a baseline urine sample, the subject ingested 104.951 g of ^2^H_2_^18^O (98.012 g of 10% AP ^18^O water and 6.939 g of 99% AP ^2^H water). The container holding the ^2^H_2_^18^O was refilled with 100 mL of tap water, which was also ingested. Subsequent urine samples were collected on days 1, 2, 4, 7 and 9 of the ride. Urine samples (∼2.5 mL) were stored in sterile screw‐cap storage vials at ambient temperature and recovered on day 11. Urine samples were then stored at 4°C until analysis. Urine samples were distilled and analysed on an Off‐Axis Integrated Cavity Output Spectrometer (OA‐ICOS; Los Gatos Instruments, Los Gatos, CA, USA) to determine the w/v ratios of H_2_^18^O/H_2_^16^O and ^2^H_2_O/H_2_O. The isotopic ratios were converted to the conventional δ notation based upon a calibrated laboratory standard. Total body water was estimated from the dilution space of ^18^O and ^2^H on day 1 using the formula:

$$N=\left[\left(N_{o}/1.007\right)+\left(N_{d}/1.043\right)\right]/2$$

as recently recommended by Speakman et al. (2021), where *N* is total body water, *N_o_* is the ^18^O dilution space and *N_d_* is the ^2^H dilution space. Total body water averaged 44.37 L (57.6% of body mass) on day 1 of the TD. Average CO_2_ production rate (rCO_2_) was estimated from the slope of the plot of the natural logarithm of the urine enrichment versus time covering the first 9 day period using the equations described by Cole and Coward (1992), with the respiratory exchange ratio assumed to be 0.92 (Miller et al., 2015).

### 2.5 Substrate oxidation

In the laboratory, we measured resting energy expenditure by indirect calorimetry (TrueOne 2400, ParvoMedics, Sandy, UT, USA) with a resting metabolic cart and ventilated hood, with the subject in a relaxed supine position, motionless but awake. The resting conditions were maintained for 30 min before the test, and steady‐state breathing was assessed for 20 min. We also performed a graded exercise test with indirect calorimetry to assess both maximal aerobic capacity (${\dot{V}}_{O_{2}\max}$) and the exercise intensity that elicited the maximal fat oxidation (FAT_max_) as described by Achten and Jeukendrup (2003). Oxygen consumption (${\dot{V}}_{O_{2}}$) and CO_2_ production (${\dot{V}}_{{\mathrm{CO}}_{2}}$) were monitored (ParvoMedics metabolic cart, Salt Lake City, UT, USA) and carbohydrate (CHO) and fat oxidation was calculated based on equations proposed by Frayn (1983). These measurements were performed before the TD and within 36 h after the race. The protocol was personalized to the subject's anticipated capacity and started with the subject cycling at 95 W and increased by 35 W every 3 min thereafter until task failure was reached. The ${\dot{V}}_{O_{2}}$ and ${\dot{V}}_{{\mathrm{CO}}_{2}}$ were measured every 15 s, and the mean ± SD of the last 45 s of each 3 min stage was plotted. Task failure was defined as an inability to maintain 90 ± 5 r.p.m. for >5 s, despite strong verbal encouragement. The time to task failure was recorded to the nearest 1 s. The ${\dot{V}}_{O_{2}}$ data were averaged in 30 s bins to identify ${\dot{V}}_{O_{2}\max}$ as the highest 30 s average ${\dot{V}}_{O_{2}}$ regardless of power output.

### 2.6 Skeletal muscle biopsy

Percutaneous muscle biopsies were taken from the vastus lateralis using a Bergstrom needle as described previously (Hafen et al., 2018). Muscle samples were separated from any fatty tissue and divided into 25–50 mg portions. In addition, a small (5–10 mg) portion was immediately placed in an ice‐cold cell buffer solution (buffer X; mM: 60 K‐MES, 35 KCl, 7.23 K_2_EGTA, 2.77 CaK_2_EGTA, 20 imidazole, 0.5 dithiothreitol, 20 taurine, 5.7 ATP, 15 mM PCr and 6.56 MgCl_2_) for analysis of respiratory capacity. Portions designated for sectioning and microscopic analysis of cross‐sectional area were mounted on a cork with tragacanth gum and frozen in isopentane cooled in liquid nitrogen. All frozen samples were stored at −80°C for analysis after completion of the study. The pre‐ and post‐TD muscle biopsies were done on opposite legs. A detailed description of the immunohistochemical methods can be found in a previously published work (Hafen et al., 2019)

### 2.7 Mitochondrial respiration

Mitochondrial respiration was measured using a high‐resolution respirometer (Oxygraph O2k; Oroboros Instruments). Muscle fibre bundles (3–5 mg) were gently teased apart to maximize surface area. Fibre bundles were weighed and placed in ice‐cold buffer X. Fibres were then permeabilized by the addition of saponin (50 μg/mL). Respiratory function was evaluated using a standard substrate–uncoupler–inhibition–titration (SUIT) protocol to assess individual components of the mitochondrial respiratory chain. Details of the SUIT protocol can be found in our previously published work (Gnaiger, 2009; Hafen et al., 2019).

### 2.8 Vascular function

Vascular endothelial function of the quadriceps femoris was assessed with passive leg movement‐induced hyperaemia. Specific details of the method have been published by our group previously (Gifford & Richardson, 2017; Hafen et al., 2019). Briefly, the participant's leg was moved passively through a 90° range of motion at a rate of 1 Hz, while the hyperaemic response was measured with Doppler ultrasound (General Electric Medical Systems, Milwaukee, WI, USA) of the common femoral artery, and the mean arterial blood pressure response was measured with finger photoplethysmography (CNAP; CNSystems, Graz, Austria). The magnitude of the flow and conductance responses were quantified as the peak and total area under the curve above baseline.

## 3 RESULTS

### 3.1 Physical, body composition and blood characteristics

**FIGURE 1:**
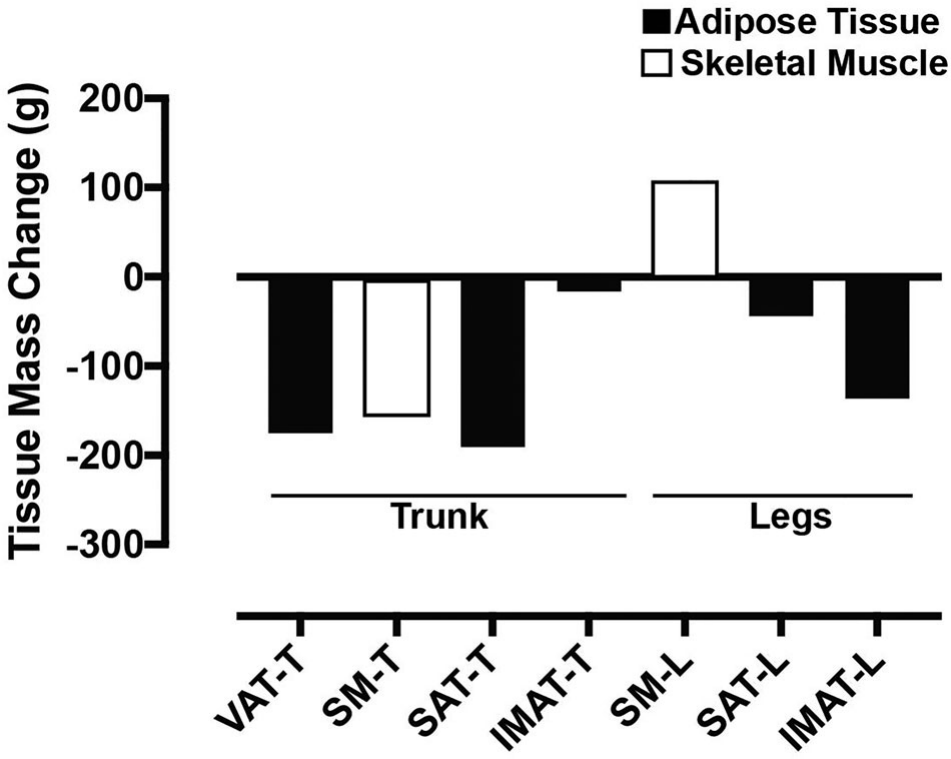
Changes in abdominal and leg tissue mass pre‐ and post‐Tour Divide. Absolute changes in adipose tissue depots and skeletal muscle were quantified by MRI. Abbreviations: IMAT‐L, intramuscular adipose tissue legs; IMAT‐T, intramuscular adipose tissue trunk; SAT‐L, subcutaneous adipose tissue legs; SAT‐T, subcutaneous adipose tissue trunk; SM‐L, skeletal muscle legs; SM‐T, skeletal muscle trunk; VAT‐T, vascular adipose tissue trunk.

The 47‐year‐old male took 16 days 7 h and 45 min to complete the route, cycling an average of 253 ± 47.6 km/day. The subject rode for 16.8 ± 2.9 h/day and slept for 5.5 ± 1.4 h/day on average. There was no change in weight or total body water (Table 1). Reductions in abdominal and appendicular adipose tissue depots contrasted with a subtle increase in skeletal muscle mass in the legs (Table 1; Figure 1). Resting energy expenditure was increased post‐TD (Table 2). Some blood biomarkers were outside of normal ranges post‐TD, including high‐density lipoprotein, alanine transaminase, aspartate aminotransferase and blood urea nitrogen (Table 3).

**TABLE 1:** Body mass and composition.

Parameter	Pre‐TD	Post‐TD	Change
Total body mass, kg	76.7	76.7	0
Total body water, kg	34.5	35.0	0.5
Visceral fat mass, kg	1.17	0.99	−0.18
Abdominal subcutaneous fat mass, kg	1.09	0.90	−0.19
Abdominal muscle mass, kg	4.61	4.46	−0.15
Abdominal intermuscular fat, kg	0.05	0.04	−0.01
Leg subcutaneous fat mass, kg	3.07	3.02	−0.05
Leg muscle mass, kg	17.8	17.9	0.01
Leg intermuscular fat mass, kg	1.41	1.28	−0.13

**TABLE 2:** Indirect calorimetry and exercise test results.

Parameter	Pre‐TD	Post‐TD	Change
Maximal O_2_ uptake, mL/kg/min	59.6	59.6	0.00
Resting energy expenditure, kcal/day	1882	2011	6.41

**TABLE 3:** Serum biomarkers.

Biomarker	Pre‐TD	Post‐TD
Triglycerides, mg/dL	44	73
High‐density lipoprotein, mg/dL	73	77
Low‐density lipoprotein, mg/dL	100	103
Albumin, g/dL	4.6	3.5
Alanine transaminase, U/L	18	68
Alkaline phosphatase, U/L	50	38
Aspartate aminotransferase, U/L	27	103
Sodium, mmol/L	143	142
Potassium, mmol/L	4.4	4.9
Chloride, mmol/L	105	106
Glucose, mg/dL	101	104
Bloodurea nitrogen, mg/dL	27	26
Creatinine, mg/dL	1.0	0.8
Calcium, mg/dL	9.0	9.6

### 3.2 Substrate utilization during exercise

**FIGURE 2:**
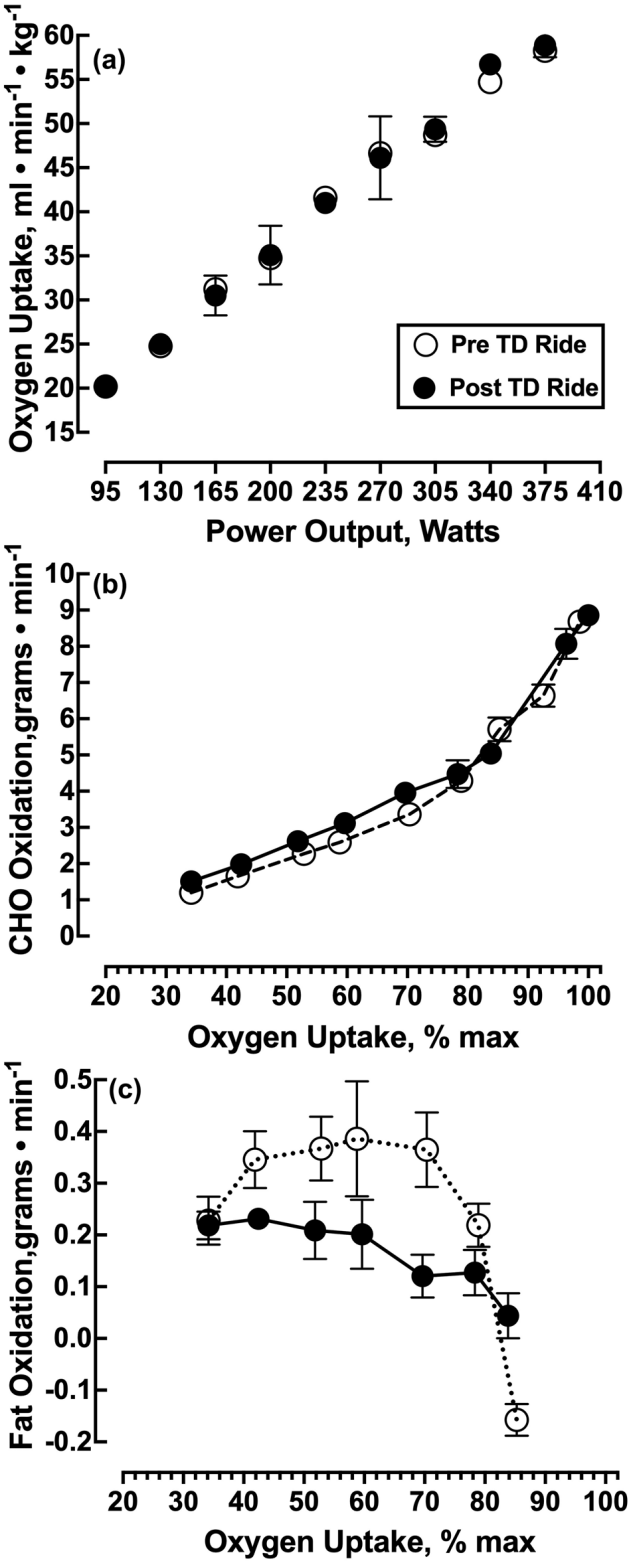
Oxygen uptake and substrate oxidation during graded exercise test pre‐ and post‐TD. Data represent the mean ± SD of the last 45 s of each 3 min stage (three values). Abbreviations: CHO, carbohydrate; TD, Tour Divide.

The ${\dot{V}}_{O_{2}\max}$ during a graded exercise test (Table 3) did not change pre‐ to post‐TD, as indicated by no change in ${\dot{V}}_{O_{2}\max}$ (Table 2) or power output at maximal effort (Figure 2a). Carbohydrate oxidation (Figure 2b) as a function of ${\dot{V}}_{O_{2}\max}$ was different pre‐ to post‐TD, and there were small differences in carbohydrate oxidation during the graded exercise test between 60% and 90% ${\dot{V}}_{O_{2}\max}$. The average FAT_max_ was reduced from 58.8% ${\dot{V}}_{O_{2}\max}$ pre‐TD to 42.3% ${\dot{V}}_{O_{2}\max}$ post‐TD (Figure 2c).

### 3.3 Energy expenditure

The average TEE estimated from ^2^H_2_^18^O measurements averaged 25.53 MJ/day (6100 kcal/day) for days 1–9 (Table 4). The dietary recall data indicated that the subject ingested ∼26.70 ± 5.17 MJ/day (6377 ± 1237 kcal/day) during the 15 days of riding.

**TABLE 4:** Tour Divide ride details and total energy intake and expenditure.

Date	Day	Destination	Ride time (h)	Distance (km)	Average elevation gained (m)	Diet recall (MJ/day)	Mean total energy intake (MJ/day)
8 June 2018	1	Banff, AB (start) to Fernie, BC	14.5	257	1641	19.89	25.53
9 June 2018	2	Fernie, BC to Sondressen, MT	17.5	266	1275	19.15	
10 June 2018	3	Sondressen, MT to Holland Lake, MT	17	248	1150	25.26	
11 June 2018	4	Holland Lake, MT to Helena, MT	18	253	1406	29.37	
12 June 2018	5	Helena, MT to Wise River, MT	17	204	1748	29.42	
13 June 2018	6	Wise River, MT to Lakeview, MT	18	299	1982	21.73	
14 June 2018	7	Lakeview, MT to Togwotee, WY	18	259	2034	28.35	
15 June 2018	8	Togwotee, WY to Boulder, WY	14	192	2177	21.03	
16 June 2018	9	Boulder, WY to Wamsutter, WY	15.5	274	2627	19.40	
17 June 2018	10	Wamsutter, WY to Steamboat Springs, CO	17.5	211	2051	33.40	
18 June 2018	11	Steamboat Springs, CO to Breckenridge, CO	14.5	227	2375	32.24	
19 June 2018	12	Breckenridge, CO to Cochepota Lakes, CO	17.5	267	2799	30.62	
20 June 2018	13	Cochepota Lakes, CO to Platoro, CO	15	214	2868	28.58	
21 June 2018	14	Platoro, CO to Cuba, NM	19	348	2674	31.74	
22 June 2018	15	Cuba, NM to Grants, NM	10	198	2140	33.82	
23 June 2018	16	Grants, NM to CDT Alt Sapillo, NM	23	357	2206	28.04	
24 June 2018	17	CDT Alt Sapillo, NM to Antelope Wells, NM	12	235	1926	21.77	

### 3.4 Skeletal muscle mitochondrial respiration

**FIGURE 3:**
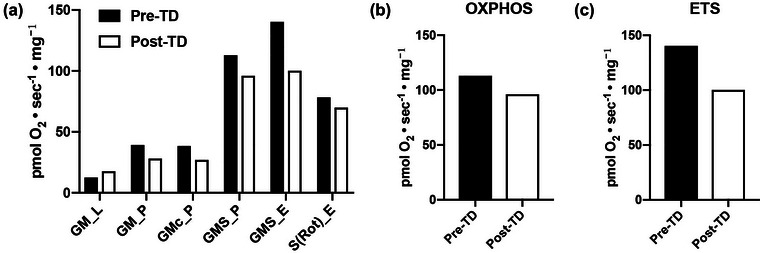
Muscle oxidative phosphorylation (OXPHOS) and electron transport system (ETS) capacity pre‐ and post‐Tour Divide (TD). (a) Oxygen flux following the addition of glutamate + malate (GM; L), + ADP (GMp), + succinate (GMSp), + FCCP (GMSe). (b) Maximal coupled respiration of permeabilized muscle fibres is an indication of mitochondrial OXPHOS capacity. (c) Maximal uncoupled respiration of permeabilized muscle fibres is an indication of ETS capacity.

To assess mitochondrial function, we performed high‐resolution respirometry on permeabilized muscle fibres from samples pre‐ and post‐TD using a standard SUIT protocol. Given that breakdown of substrates drives respiration through the first two mitochondrial proteins (complexes I and II), the substrates were added in a stepwise fashion to ensure that both complexes were contributing maximally. Maximal coupled respiratory capacity (OXPHOS) was reached after the addition of glutamate + malate + ADP for complex I (GMp; PI), and succinate for complex II (GMSp; PI+II). After the attainment of maximal coupled respiration, oxidative phosphorylation was uncoupled by the titration of FCCP in order to assess maximal uncoupled respiration (GMS_E; Figure 3a). The OXPHOS (Figure 3b) and electron transport system (ETS; Figure 3c) flux were both decreased post‐TD, by 14.9% and 28.5%, respectively.

### 3.5 Skeletal muscle histology

**FIGURE 4:**
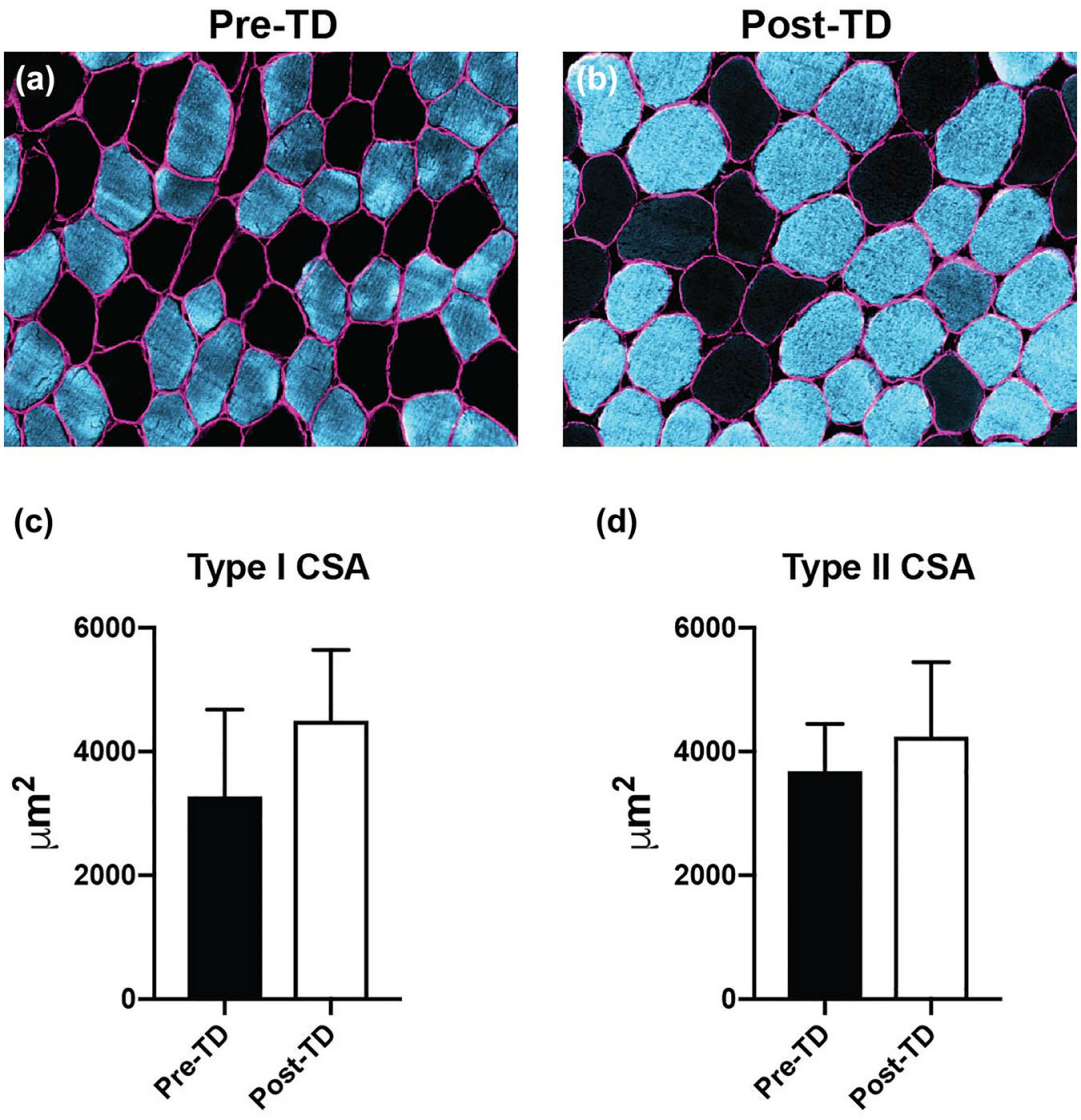
Muscle fibre cross‐sectional area (CSA) pre‐ and post‐TD. (a, b) Representative images of muscle cross‐sections from biopsy samples taken pre‐TD (a) and post‐TD (b). Pink = dystrophin (myofibre membrane); blue = myosin heavy chain type I; absence of stain for myosin heavy chain type I indicates type II fibres (black). (b, c) Measurement of type I (c) and type II (d) muscle fibre CSA pre‐ and post‐TD. Abbreviations: CSA, cross‐sectional area; TD, Tour Divide.

There was no direct histological evidence of muscle damage, as indicated by necrotic fibres or discontinuous dystrophin staining patterns post‐TD (Figure 4a,b). Both type I and type II fibre cross‐sectional area were increased post‐TD, by 37.3% (3285.6 vs. 4510.9 μm^2^) and 15.0% (3697.6 vs. 4252.8 μm^2^), respectively (Figure 4c,d). Additionally, the ratio of type I to type II fibres increased from pre‐ (type I = 56.1%, type II = 43.9%) to post‐TD (type I = 66.2%, type II = 33.8%).

### 3.6 Vascular function

**FIGURE 5:**
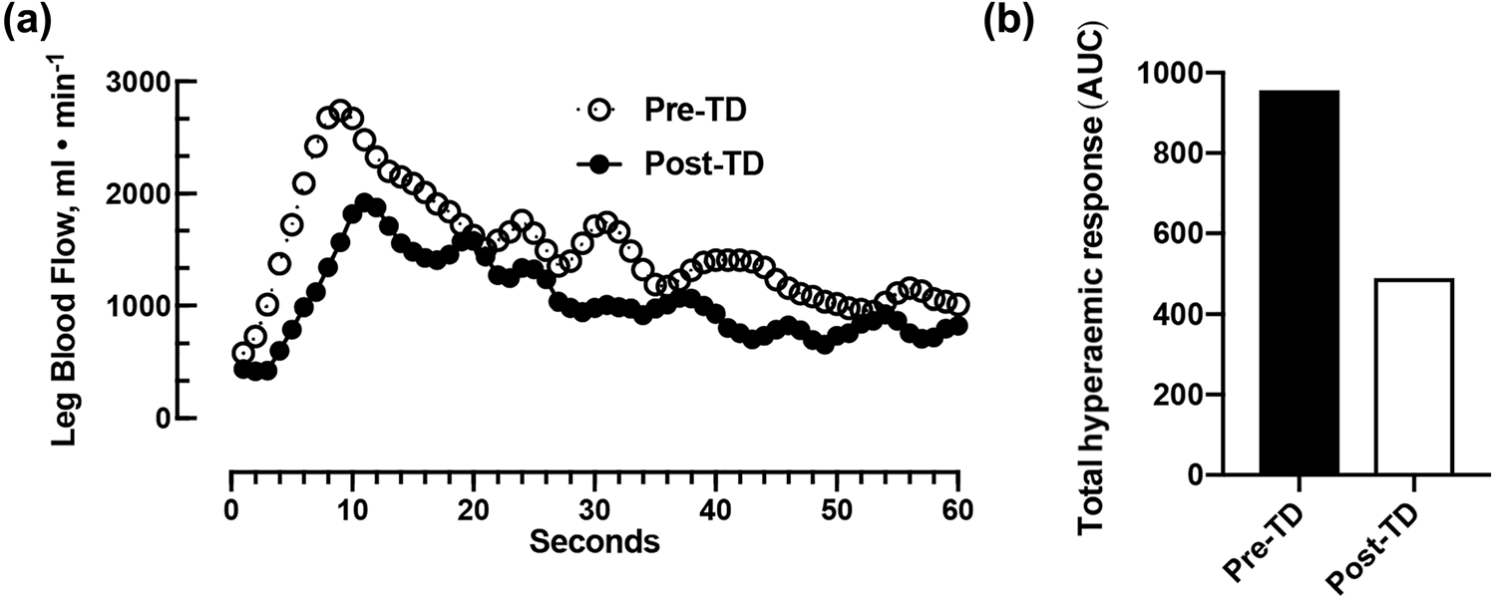
Vascular endothelial function pre‐ and post‐TD. (a) Illustration of the blood flow response to PLM pre‐ and post‐Tour Divide. (b) Total hyperaemic response (i.e., AUC) to PLM, which is a marker of endothelial function, before and after the race. Abbreviations: AUC, area under the curve; PLM, passive leg movement; TD, Tour Divide.

Overall, there was a marked decrease in the peak (pre‐TD, 2841 mL/min; post‐TD, 2014 mL/min; 29% decrease) and total (pre‐TD, 959 mL; post‐TD, 491 mL; 49% decrease) leg blood flow responses during the passive leg movement post‐TD (Figure 5), indicating compromised vascular endothelial function after the completion of the race. This change appears to be driven primarily by changes in resistance artery function, because the peak change in vascular conductance (pre‐TD, 27.6 mL/min/mmHg; post‐TD, 20.8 mL/min/mmHg; 25% decrease) and total change in vascular conductance (pre‐TD, 10.7 mL/mmHg; post‐TD, 6.7 mL/mmHg; 38% decrease) were markedly reduced post‐TD. Importantly, the observed changes in vascular function were accompanied by large changes in peak blood velocity (pre‐TD, 41.4 cm/s; post‐TD, 28.4 cm/s; 31% decrease), but little to no change in resting femoral artery diameter (pre‐TD, 1.10 cm; post‐TD, 1.10 cm; 0% change), resting velocity (pre‐TD, 9.6 cm/s; post‐TD, 9.6 cm/s; 0% change) and resting flow (pre‐TD, 549 mL/min; post‐TD, 547 mL/min; 0.03% decrease).

## 4 DISCUSSION

In this case study, we took a broad and comprehensive approach to investigate the systemic, cardiovascular and skeletal muscle changes that accompany a unique physiological stress. Several interesting and potentially important observations were made: (1) muscle mitochondrial and vascular function were markedly compromised, despite no apparent decrease in exercise performance; (2) vastus lateralis muscle appears to have both increased in mass and shifted towards a more oxidative phenotype in a relatively short period of time (16 days); and (3) TEE generally matched our estimation of caloric intake. Collectively, the data indicate the remarkable capacity of the cardiovascular and muscular systems to perform under an extreme long‐term physical stress, despite some evidence of compromised oxygen delivery and utilization systems.

One of our more remarkable findings was the marked decrease in both mitochondrial OXPHOS and ETS capacity. Biopsy studies in the ultra‐endurance literature are rare, yet there is some precedent for such changes after exercise stress. For example, when Konopka et al. (2017) used similar methods to assess vastus lateralis mitochondrial function 36 h after the completion of a 5 day ultra‐endurance mountain bike race; they likewise found post‐ride decreases of OXPHOS and ETS capacity of ∼30%. It remains uncertain why muscle respiratory deficits are observed after ultra‐endurance exercise. Konopka et al. (2017) argued that because endurance training generally results in a substrate preference shift towards fatty acid oxidation, decreases in respiratory capacity might simply be an artefact of the carbohydrate‐dependent assay. However, this argument is not supported by the present study, wherein our subject's substrate preference shifted towards CHO during exercise post‐TD. It is noteworthy that mitochondrial function has been assessed only in highly trained athletes shortly (within 3 days) following the cessation of the ultra‐endurance effort. Layec et al. (2018) showed that OXPHOS capacity was reduced by ≤40% immediately after a 5 km cycling time trial to exhaustion. Perhaps our measures, taken only 36 h after the completion of the event, reflect this more acute effect.

Similar to muscle mitochondrial respiratory capacity, leg vascular endothelial function was impaired post‐TD. As described previously (Gifford & Richardson, 2017), the magnitude of the increase in blood flow during passive knee extension and flexion is highly dependent upon the bioavailability of nitric oxide within the vasculature of the leg, making the magnitude of hyperaemia an index of vascular endothelial health. As illustrated in Figure 5, the subject exhibited a 48% decrease in the total hyperaemic response (i.e., area under the curve) to passive leg movement post‐TD, suggesting that the NO bioavailability was markedly reduced post‐TD. This change is substantial and on par with decreases we have previously seen after 10 days of leg immobilization (Hafen et al., 2019). Previous studies examining the impact of acute exercise (0.5–2 h) on endothelial function have reported a biphasic response with endothelial function, assessed by flow‐mediated dilatation, exhibiting an initial decrease in the first 30 min after exercise that typically normalizes after 2 h (Dawson et al., 2013). Notably, the subject in the current study had rested for 36 h at the time of measurement. Clearly, the duration of exercise in the present study is much longer (average of ∼16 h/day for 17 days) than a single acute bout of exercise. Therefore, it might be more appropriate to compare the change in endothelial function with adaptations elicited by 2 weeks of endurance training. Contrary to the results of the present study, endothelial function, assessed by flow‐mediated dilatation, is typically augmented in response to 2 weeks of training, after which structural modifications in the artery can contribute to a normalization of flow‐mediated dilatation by 8 weeks of training (Green et al., 2004; Tinken et al., 2008). The present data are in agreement with data from Jee and Jin (2012), who reported significant increases in markers of endothelial dysfunction following an ultramarathon race. At this point, it is unclear how long the endothelial function remains decreased or whether this is attributable to damage, dysfunction or remodelling. Remarkably, however, despite marked decreases in both vascular and mitochondrial function, the subject experienced no decrease in exercise performance from pre‐TD to post‐TD. This suggests that highly trained athletes possess an energetic reserve that allows for high levels of function in suboptimal energetic conditions.

As expected, our subject lost fat mass in both appendicular and visceral compartments, but unexpectedly, our lower‐body MRI analysis showed that leg skeletal muscle mass was maintained or even slightly increased from pre‐ to post‐TD. Corroborating these data, vastus lateralis myofibre cross‐sectional area increased. Given the supposed catabolic environment, stimulus of exercise and relatively short time frame, it seems unlikely that hypertrophy of the leg musculature could have occurred over the course of the 16 day period of the race. Although cycle endurance training is capable of inducing vastus lateralis hypertrophy (Fry et al., 2014), we know of no other published studies that have demonstrated myofibre hypertrophy with <16 days of endurance cycling training. However, we must note that the pre‐TD measures were obtained >1 month before the start of the race; therefore, it is possible that the subject could have increased his mass during the lead up to the race while being in a more anabolic, fed state. Nevertheless, it is noteworthy that the ultra‐endurance stimulus, at the very least, did not result in a loss of leg skeletal muscle mass.

In this study, we were able to measure TEE reliably only during the first 9 days of riding. However, the average distance, ride time and altitude were not different in days 1–9 versus days 10–17. Our estimate of total energy intake was also similar for the two intervals, and the subject maintained body weight, indicating that he was in energy balance. Thus, it is likely that TEE was not significantly different during days 10–17. Our estimates of TEE are also in line with other ultra‐endurance studies. For example, Plasqui et al. (2019) recently published a report detailing the TEE of seven cyclists during the Giro d'Italia (24 day, 3445 km bicycle race). Using a similar approach, they estimated a TEE of between 29.9 and 32.3 MJ/day (6904 and 7717 kcal/day). Likewise, a study on two men crossing Antarctica over the course of 90 days, who maintained a similar pattern of ∼10 h of exercise per day over 2300 km, reported TEE values ranging from 23.2 to 47.7 MJ/day (5568–11,639 kcal/day) (Stroud et al., 1997). Collectively, the TEE, total energy intake and body weight data reported herein suggest that the subject was at or close to energy balance for the duration of the event.

Overall, despite multiple limitations, this case study brings to light several new observations in the highly under‐studied area of extreme human performance. Although some notable acute deficiencies were found in muscle mitochondrial and vascular function, most general prognosticators of health (i.e., blood biomarkers, morphological measures and performance indicators) were found to be affected minimally by the bicycle race. Although it is impossible to speculate regarding the long‐term consequences, at the very least, our data suggest a remarkable plasticity and tolerability for the extreme physical stress imposed by multi‐day ultra‐endurance events in a trained athlete.

## AUTHOR CONTRIBUTIONS

Robert D. Hyldahl, Jayson R. Gifford, Lance E. Davidson, Chad R. Hancock, and Gary W. Mack conceived of the work, aquired data, drafted the manuscript and approved the final version of the manuscript. Paul S. Hafen, and Allen C. Parcell acquired data, drafted the manuscript and approved the final version of the manuscript. All authors agree to be accountable for all aspects of the work. All persons designated as authors qualify for authorship, and all those who qualify for authorship are listed.

## FUNDING INFORMATION

None.

## CONFLICT OF INTEREST

The authors declare no conflicts of interest.
